# Methotrexate Induced Pancytopenia

**DOI:** 10.1155/2014/679580

**Published:** 2014-05-27

**Authors:** Fernando Gonzalez-Ibarra, Sahar Eivaz-Mohammadi, Shiri Surapaneni, Hazem Alsaadi, Amer K. Syed, Simon Badin, Valentin Marian, Mazhar Elamir

**Affiliations:** ^1^Department of Internal Medicine, Mount Sinai School of Medicine, Jersey City Medical Center, 355 Grand Street, Jersey City, NJ 07302, USA; ^2^Department of Internal Medicine, Jersey City Medical Center, St. George's University School of Medicine, Jersey City, NJ 07302, USA; ^3^Laureate National Institute of Medicine, Jersey City Medical Center, 355 Grand Street, Jersey City, NJ 07302, USA; ^4^Department of Hematology, Mount Sinai School of Medicine, Jersey City Medical Center, Jersey City, NJ 07302, USA; ^5^Department of Rheumatology, Mount Sinai School of Medicine, Jersey City Medical Center, Jersey City, NJ 07302, USA

## Abstract

The well-reported methotrexate (MTX) toxicities are based on the duration and cumulative dosing of drug. The typical toxicities can be predicted by the timing of drug administration, where mucositis occurs as an earlier effect, while myelosuppression and the sequelae of pancytopenia occur later after MTX administration. Despite these well-known toxicities, low dose MTX therapy can become problematic, in particular with the elderly, who are at a greater risk for significant myelosuppression. We present a case of a 73-year-old female with pancytopenia causing severe neutropenia, mucocutaneous bleeding, and bruising and requiring intravenous antibiotic therapy and limited transfusion dependence as a result of low dose daily MTX for rheumatoid arthritis.

## 1. Introduction

MTX is a widely used drug most commonly used in the treatment of various malignancies and autoimmune disorders, including rheumatoid arthritis and elective abortions. It is an inhibitor of cellular proliferation. As such, cells with the highest turnover or reduced half-life are most susceptible to its effect. As a consequence, when a patient's oral epithelial cells are affected, mucositis develops. Via the same mechanism, cytopenia leads to increased bleeding, easily bruising, macrocytic erythrocytes, and an increased risk of infections [1–3].

## 2. Case Report 

A 73-year-old African American female with a past medical history of CAD, CVI, hypertension, and rheumatoid arthritis presents to the emergency room with epistaxis and gingival bleeding which initially began as a mild oral mucositis but progressed to odynophagia. She also experienced increased bruising for the last couple of weeks. These bruises progressed to open bleeding ulcerations with minimal trauma. Despite this, the patient had no prior hematological or oncological pathologies. Her home medications included aspirin (81 mg), carvedilol, enalapril, folic acid, furosemide, clopidogrel, MTX, and simvastatin. Her rheumatologist had recently changed her dosage of MTX from 7.5 mg weekly to 2.5 mg daily approximately two months prior due to worsening of her rheumatoid arthritis symptoms. Apparently the patient did not have a proper outpatient monitor of CBC, creatinine, or liver function tests after MTX dose was increased. Vital signs are pulse (68 bpm), respiratory rate (18/minute), blood pressure (152/85), and temperature (98.7°C). Her physical examination is evident for multiple petechiae scattered in the upper body. On admission, patient is pancytopenic: WBC (2.0; differential: polys = 23%, lymphocytes = 55%, monocytes = 2%, and eosinophils = 20%), Hb (7), MCV (89), and platelets (3000). Her last CBC two months prior was WBC (7.6), Hb (10.2), and platelets (194).

The following findings were also present: BUN = 36, creatinine = 1.5, AST = 27, and ALT = 26. Chest X-ray showed multiple scattered pulmonary infiltrates. Given her near normal CBC 2 months prior, we were suspicious of MTX induced myelosuppression. She was immediately started on leucovorin. A bone marrow evaluation was performed to rule out malignancy and myelodysplasia; however, no bone marrow could be aspirated and the biopsy attempt was not tolerated by the patient.

The patient was transfused with blood products including packed red blood cells, platelets, and fresh frozen plasma. She was empirically started on broad spectrum antibiotics for neutropenic fever and MTX was stopped. She improved significantly and pancytopenia resolved with almost normal hematologic parameters in less than a week of followup.

## 3. Discussion

MTX is a folate antagonist used in the treatment of various malignancies, autoimmune disorders, and abortion. It is transported into cells by an active cellular uptake and an active efflux transporter. Once in the cell, MTX inhibits dihydrofolate reductase (DHFR), an enzyme responsible for the conversion of dihydrofolate (DHF) to tetrahydrofolate (THF) [4, 5]. Consequently, there is a reduction in thymidylate and purine biosynthesis. DNA synthesis eventually halts and cells can no longer replicate [4]. Polyglutamination of this drug prolongs its intracellular presence [6, 7]. Hence, cells with the capability of effective polyglutamination such as leukemic myeloblasts, synovial macrophages, lymphoblasts, and epithelia are more susceptible to this medication [6, 7]. On the contrary, an increase in polyglutamination results in increased risk of toxicity as a result of direct prolonged intracellular exposure. Myeloid lineage megakaryocytes and epithelial polyglutamination increase the intracellular concentration of MTX and consequently patients may experience ulcers and bleeding as in this case. The same is true for WBC and RBC, which may manifest as infections and macrocytic anemia, also present in this case [8].

In addition to antifolate pathway, MTX also works on the adenosine pathway with important anti-inflammatory effects. Inhibition of transformylase (ATIC) by MTX-PG leads to accumulation of 5-aminoimidazole-4-carboxamide ribonucleotide (AICAR) and ultimately leads to increased levels of adenosine. Adenosine is a potent inhibitor of inflammation and induces vasodilation. Adenosine's anti-inflammatory effects include regulation of endothelial cell inflammatory functions, including cell trafficking. This effect of MTX does not seem to be affected by folate supplementation [9].

Resistance to MTX is variable and can include increased DHFR and efflux, as well as decreased polyglutamination and uptake. MTX may be administered orally in low doses (5–10 mg/m^2^) or parenterally in high doses (>25 mg/m^2^). The starting dose is usually 5 to 10 mg given as a single weekly dose. More frequent administration is associated with a significantly increased risk of liver toxicity. If the oral dose of MTX exceeds 15 mg, consideration should be given to splitting the dose, with each half given 6 to 12 hours apart, for improved bioavailability. The dosage of MTX can be escalated gradually, usually every 4 to 8 weeks up to 25 mg/week [10, 11]. Most MTX is excreted in the urine within the first 12 hours after administration, except for MTX-PG. MTX and metabolites not only are excreted by the kidney by glomerular filtration and proximal tubular secretion but also undergo distal tubular reabsorption. The estimated median half-life of elimination of MTX-PG is 3.1 weeks (ranging from 0.94 to 4.1 weeks), and MTX-PG is undetectable at 15 weeks. Serum half-life is 10 hrs, but tissue half-life of MTX-polyglutamate is more than 3 weeks. At low doses, MTX can be administered either orally or parenterally (subcutaneous or intramuscular), and absorption is rapid, peaking from 1 to 2 or from 0.1 to 1 hour, respectively. The absorption of low-dose oral and parenteral MTX (<15 mg/wk) is roughly equivalent, but once the oral dose exceeds 15 mg/wk, absorption diminishes by as much as 30% [10, 12–15]. Although not prospectively studied in RA patients receiving long-term MTX treatment, the parenteral route should have diminished potential for hepatotoxicity. This effect has been seen in a retrospective study wherein more elevations in transaminases were noted when oral MTX was administered to the same individuals versus when given parenterally [16].

Adverse effects are of vital importance as they can be quickly progressive and fatal. The main ones include myelosuppression as in this case, pneumonitis, hepatotoxicity, and gastrointestinal toxicity [2]. Early toxicity is oral mucositis while severe toxicity includes bleeding, as with our patient. Rash and neurotoxicity (via the intrathecal route) are also observed. Toxicity can be increased in renal impairment or reduced renal blood flow, as with NSAIDs use [17]. In fact, MTX is contraindicated in any patient with eGFR <30 mL/min [18]. It is advised that routine blood count be performed every four to eight weeks [1]. Concomitant administration of folic acid (1 to 3 mg/day) decreases the frequency of toxicities, including mucositis, nausea, hematologic abnormalities, and liver enzyme elevations, without seeming to interfere with clinical efficacy [8, 19].

This case is extraordinary due to an unfortunate chain of events that led to severe toxicity. Despite initial concerns, when given once a week in doses used for rheumatic diseases and monitored correctly, MTX is very well tolerated. Five important events need to be clearly pointed out as critical when we care for patients with rheumatic diseases on therapy with MTX: (1) patients older than 65 years represent a special subset at particular high risk for toxicity (our patient is 75 years old). There are differences among the pharmacokinetic profiles in this group of patients, including drug distribution as a result of decreases in end-organ blood flow and lean body mass, decreased hepatic drug metabolism, and decreased renal drug excretion. The serum creatinine may be a misleading measure of renal function in older patients owing to an overall reduction in lean muscle mass. Dosing recommendations are as follows: initial doses should be around 5 to 7.5 mg/wk and should not exceed 20 mg/wk. Dosage adjustments for CrCl are as follows: for a CrCl of 61 to 80 mL/min, reduce the dose by 25%; for a CrCl of 51 to 60 mL/min, reduce the dose by 30%; for a CrCl of 10 to 50 mL/min, reduce the dose by 50% to 80%; and for a CrCl less than 10 mL/min, avoid use [20]; our patient had a GFR <40 and her dose should have been reduced by 50%. (2) MTX should not be given more frequent than once a week due to increased toxicity risk (our patient received daily dosing); alternatively parenteral dosing could be used for better bioavailability and less GI and liver toxicity. (3) MTX dose should be gradually increased by no more than 2.5 mg every 1 to 2 weeks; our patient dose was increased from 7.5 mg/week to 17.5 mg/week. (4) Monitoring for toxicity should be done every 2 to 4 weeks for the first 3 months of therapy; our patient had no blood work done for 2 months after the dose was increased before she presented to ER.

Bone marrow toxicity, in most cases, is dose dependent and responds to folic acid administration. Pancytopenia, leukopenia, anemia, and thrombocytopenia can occur but are rare. In a review by Gutierrez-Urena and associates, clinically significant pancytopenia was found in 1% to 2% of RA patients on MTX therapy [2]. The mucocutaneous toxicities of MTX, which have been reported to occur in up to one-third of patients, are dose dependent and respond to folate replacement.

## 4. Conclusions 

It is of utmost importance that primary care physicians are aware of these complications and recommendations, because the majority of these serious complications can be detected on time and even prevented. Patients on MTX therapy should be regularly monitored with liver function tests and CBC to identify myelosuppression and avoid the sequelae of pancytopenia. Renal function must also be monitored as this drug uses mainly the kidneys for excretion.
